# Editorial: Extremozymes: characteristics, structure, protein engineering and applications

**DOI:** 10.3389/fmicb.2024.1423463

**Published:** 2024-05-08

**Authors:** Hitarth B. Bhatt, Rajesh K. Sani, Mohammad Ali Amoozegar, Satya P. Singh

**Affiliations:** ^1^Department of Microbiology, Faculty of Science, Atmiya University, Rajkot, Gujarat, India; ^2^Department of Chemical and Biological Engineering, South Dakota School of Mines and Technology, Rapid City, SD, United States; ^3^Extremophiles Laboratory, Department of Microbiology, School of Biology, College of Science, University of Tehran, Tehran, Iran; ^4^Department of Biosciences, Saurashtra University, Rajkot, Gujarat, India

**Keywords:** extremophiles, extremozymes, enzyme engineering, structure-function relationship, enzyme optimization, enzyme purification and characterization, enzyme applications, enzyme immobilization

Enzymes are indispensable biological catalysts that play pivotal roles in myriad physiological processes. They accelerate chemical reactions without being consumed in the process (Liu and Kokare, 2023). Overall, the versatility and efficiency of enzymes make them indispensable in diverse fields, concerning processes, products, and cellular and environmental sustainability.

Extremozymes are obtained from organisms that thrive in extreme environmental conditions, including high or low temperatures, acidic or alkaline pH levels, and high salinity (Sarmiento et al., 2015). These enzymes exhibit unique characteristics of stability and function in harsh conditions, which make them invaluable in various applications and industrial processes (Ghattavi and Homaei, 2023; Kikani et al., 2023; Rai et al., 2024). Extremozymes have diverse application avenues, including bioremediation, pharmaceuticals, detergents, food and beverage, and biofuel production (Mesbah, 2022).

To harness extremozymes for sustainable industrial processes, it is crucial to delve deeper into the microbial diversity of different extreme environments and uncover novel enzymes having combinations of unique properties (Bhatt and Singh, 2022). With the increasing focus on sustainability, extremozymes offer a promising route to efficient biocatalysis in harsh conditions, minimizing energy use, waste, and environmental harm. Furthermore, advances in X-ray crystallography and cryo-electron microscopy are key for detailing extremozyme structures at atomic levels. Techniques like directed evolution and rational design have improved extremozymes' stability and efficiency (Nakhaee et al., 2018; Rigoldi et al., 2018; Liu et al., 2019). An interdisciplinary approach incorporating bioinformatics, synthetic biology, and materials science will further enhance the application of extremozymes in addressing global challenges.

This Research Topic aimed to delve into emerging insights regarding the structure and function of extremozymes. For this Research Topic, seven articles reflecting diverse aspects of the theme were published. These involved identifying enzymes capable of operating in extreme conditions, unraveling their adaptation mechanisms and structure-function relationships, and enhancing catalytic properties through protein engineering and innovative applications.

Shi et al. present a thorough examination of thermostable DNA ligases sourced from hyperthermophilic bacteria and archaea, spotlighting their structural and biochemical characteristics. The review delves into a comparative analysis between thermostable DNA ligases and their non-thermostable counterparts, elucidating the differences between bacterial and archaeal variants. Despite significant progress, the intricate structures of thermostable DNA ligases continue to pose challenges. The manuscript also explores potential pathways for enhancing the features and applications of thermostable DNA ligases through modification.

Karthik et al. unveiled the potential bioactive compounds from *Streptomyces tauricus* of mangrove ecosystems, and their impact on inhibiting prostate cancer PC3 cells. After optimized growth conditions for *S. tauricus*, the intracellular extract was purified and analyzed by GCMS and LCMS revealing low molecular weight peptides including Tryprostatin B, Fumonisin B1, Microcystin LR, and Surfactin C. The findings suggest the capacity of *S. tauricus* to generate bioactive peptides with dual antimicrobial and anticancer properties.

In another contribution by Røyseth et al., characteristics of the novel C11 protease globulin from uncultivated *Archaeoglobales* in the Soria Moria hydrothermal vent system on the Arctic Mid-Ocean Ridge have been described. The sequence comparison in the MEROPS-MPRO database revealed its correlation with C11-like proteases of the human gut bacteria. Through recombinant expression in *Escherichia coli*, they evaluated the wild-type zymogen and 13 mutant variants to reveal the maturation and function of the enzyme. Globupain stands out for its high thermostability and activity in low pH, and high reducing conditions, establishing its suitability in industrial and biotechnological applications.

The potential applications of TrLipE, a thermophilic lipase from *Thermomicrobium roseum*, have been constrained by its relatively low enzymatic activity. In their manuscript, Fang et al. detail the creation of 18 chimeras through lid swapping, which displayed higher expression levels than the wildtype TrLipE. Molecular dynamics (MD) simulations revealed increased flexibility in these chimeras. Moreover, these variants exhibited enhanced thermostability and resilience across a broad pH range compared to other thermostable lipases. Significantly, variants with single, double, or tripe substitutions demonstrated 2-3-fold faster catalysis than the wild TrL17.

Similarly, processed thermostable lipase from *Brevibacillus* sp. SHI-160 using an alcohol-salt-based aqueous two-phase system has been described by Leykun et al. The versatility of the enzyme reflected by the optimal activity at 65°C and stability in polar solvents, offers potential application prospects. Its efficient recovery, immobilization, and performance in non-aqueous media highlight its potential for cost-effective synthesis of valuable compounds.

Sun et al. have described an innovative approach, combining droplet-based microfluidics with conventional CSR for polymerase mutant site screening for enhanced salt tolerance. Substituting regular sites with conserved amino acids was projected as an effective strategy. SZ_A emerged as the most promising variant, offering improved salt tolerance, processivity, and exonuclease deficiency, ideal for nanopore sequencing. This methodology not only enhances polymerase activity for salt tolerance but also introduces a novel sequence design strategy through amino acid substitution, paving the way for biotechnological advancements and new applications.

Hu et al. successfully identified and modified the enzyme motion pathway of an α-amylase from *Geobacillus stearothermophilus*. The mutants exhibited enhanced characteristics. Particularly, the P44E mutation enhanced hydrolytic activity by 95% and catalytic efficiency by 93.8%, with an optimal temperature of 90°C. It was revealed that amino acids located in the central region of the conformational motion pathway serve as critical “hinge” positions, crucial for structural stability. It facilitated substrate entry and exit from the catalytic center. The strategic designing of the pivotal amino acids could modify enzymes with significantly improved activities and stability.

In conclusion, the research featured in this topic signifies significant advancements in biotechnology and enzyme engineering. It includes the exploration of thermostable DNA ligases, identification of bioactive compounds with potential anticancer properties, introduction of a novel protease, engineering of lipase variants for improved catalysis, and presentation of innovative approaches for polymerase activity enhancement. These studies offer valuable insights and drive progress in biotechnological research, particularly in the pursuit of novel extremozymes and refining existing properties through molecular evolution techniques.

## Author contributions

HB: Conceptualization, Writing—original draft, Writing—review & editing. RS: Writing—review & editing. MA: Writing—review & editing. SS: Conceptualization, Writing—original draft, Writing—review & editing.
